# Supplementary material for: Whole genome CRISPRi screening identifies druggable vulnerabilities in an isoniazid resistant strain of Mycobacterium tuberculosis
Source: Nat Commun. 2024 Nov 13;15:9791. doi: 10.1038/s41467-024-54072-w (PMC11560980; doi:10.1038/s41467-024-54072-w)
Supplement: Supplementary file 1 — Supplementary Information [file 41467_2024_54072_MOESM1_ESM.pdf]

Supplementary information for:

Whole genome CRISPRi screening identifies druggable vulnerabilities in an isoniazid resistant strain of *Mycobacterium tuberculosis*

XinYue Wang[1], William J Jowsey[1], Chen-Yi Cheung[1], Caitlan J Smart[1], Hannah R Klaus[1,2], Noon EJ Seeto[1], Natalie JE Waller[1], Michael T Chrisp[1], Amanda L Peterson[3], Boatema Ofori-Anyinam[4,5], Emily Strong[6], Brunda Nijagal[3], Nicholas P West[6], Jason H Yang[4,5], Peter C Fineran[1,2,7,8], Gregory M Cook[1,2,9], Simon A Jackson[1,2], Matthew B McNeil[1,2]*

[1] Department of Microbiology and Immunology, University of Otago, Dunedin, New Zealand

[2] Maurice Wilkins Centre for Molecular Biodiscovery, University of Auckland, Auckland, New Zealand

[3] Metabolomics Australia, Bio21 Institute, The University of Melbourne, Melbourne, Australia

[4] Center for Emerging and Re-emerging Pathogens; Public Health Research Institute; Rutgers New Jersey Medical School; Newark, NJ 07103; USA

[5] Department of Microbiology, Biochemistry, and Molecular Genetics; Rutgers New Jersey Medical School; Newark, NJ 07103; USA

[6] School of Chemistry and Molecular Biosciences, The University of Queensland, Brisbane, Queensland, 4072, Australia

[7] Genetics Otago, University of Otago, Dunedin, New Zealand

[8] Bio-Protection Research Centre, University of Otago, Dunedin, New Zealand

[9] School of Biomedical Sciences, Queensland University of Technology, Translational Research Institute, Woolloongabba, Queensland 4000, Australia

* Correspondence and requests should be addressed to M.M (matthew.mcneil@otago.ac.nz)

Supplementary Figures

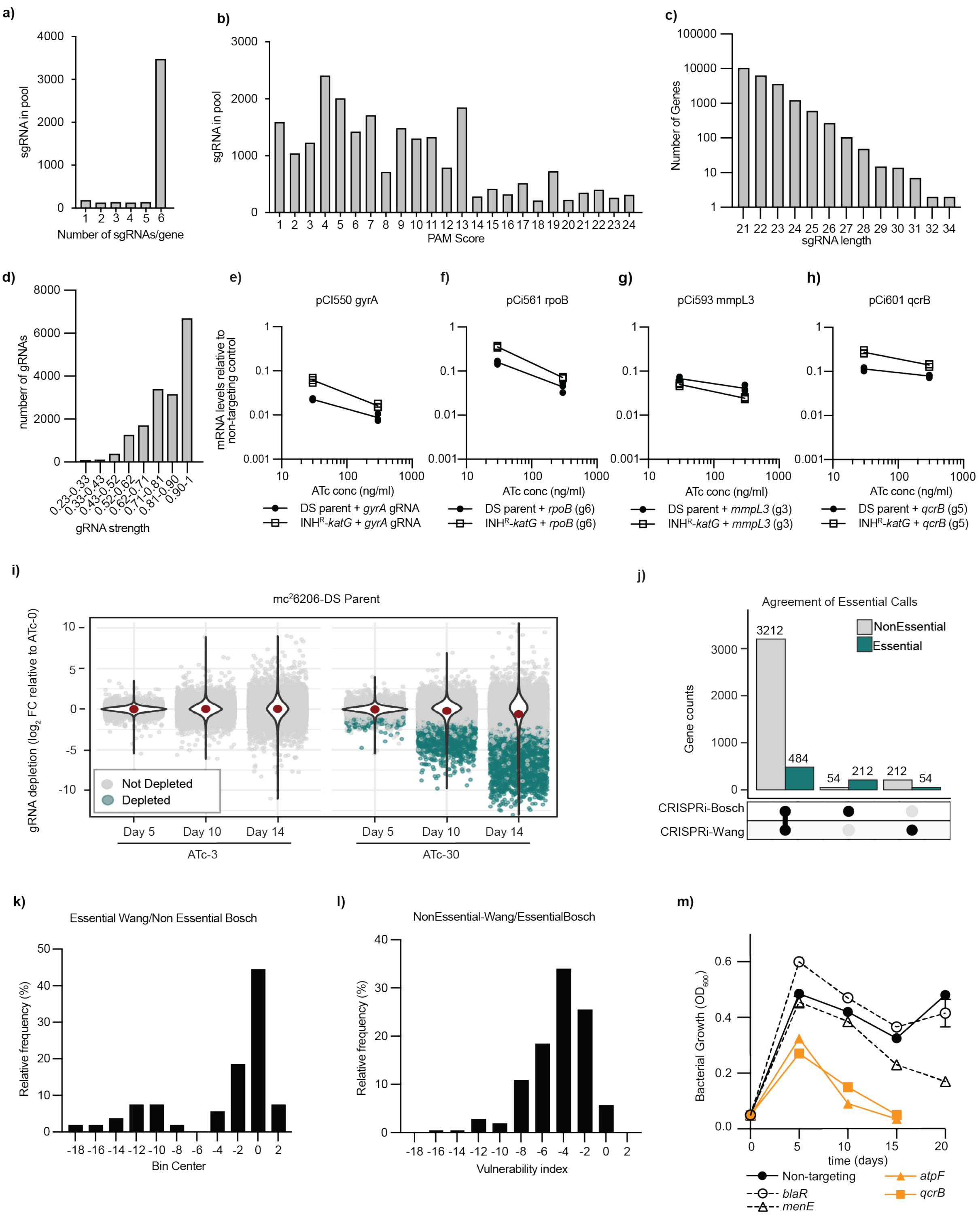

Supplementary Fig. 1| Characteristics of the constructed WG-CRISPRi library and genetic vulnerability validation: Distribution of **(a)** the number of gRNAs targeting each gene, **(b)** the predicted PAM score of each gRNA based on Rock et al 2017, **(c)** the length of gRNAs and **(d)** the predicted gRNA strength based on Bosch et al 2021. **(e-h)** qPCR results shown as the level of transcriptional repression for four candidate genes (e) *gyrA*, (f) *rpoB*, (g) *mmpL3* and (h) *qcrB*) at multiple ATc concentrations (i.e 30 and 300 ng/ml) in both the DS parent and INH^{R}-*katG* strain **(i)** The summary of gRNA

abundance at ATc-3 and ATc-30 in the *M. tuberculosis* strain $mc^2$6206 DS-parent. **(j)** The overlap in gene essentiality calls between CRISPRi-Bosch and our WG-CRISPRi study (CRISPRi-Wang). **(k-l)** Distribution of VI of target genes with discrepant essentiality predictions between CRISPRi-Bosch and CRISPRi-Wang. Genes frequencies were shown in percentage format (c) VI of disagreed target genes that we called essential but Bosch called non-essential (n = 212 unique genes) (d) VI of disagreed target genes that we called non-essential but Bosch called essential (54) **(m)** Growth kinetics of *M. tuberculosis* DS-parent expressing gRNAs that target *menE* and *blaR*, i.e. genes identified as essential in CRISPRi-Bosch, but non-essential in CRISPRi-Wang. A non-targeting gRNA is included as a negative control, whilst gRNA targeting *atpF* and *qcrB* are included as essential genes. Bacterial growth was measured by OD_{600} and back diluted 1/20 into fresh media on days 5, 10, 15 and 20. All strains were grown in 7H9-K with ATc-300. Source data are provided as a Source Data file.

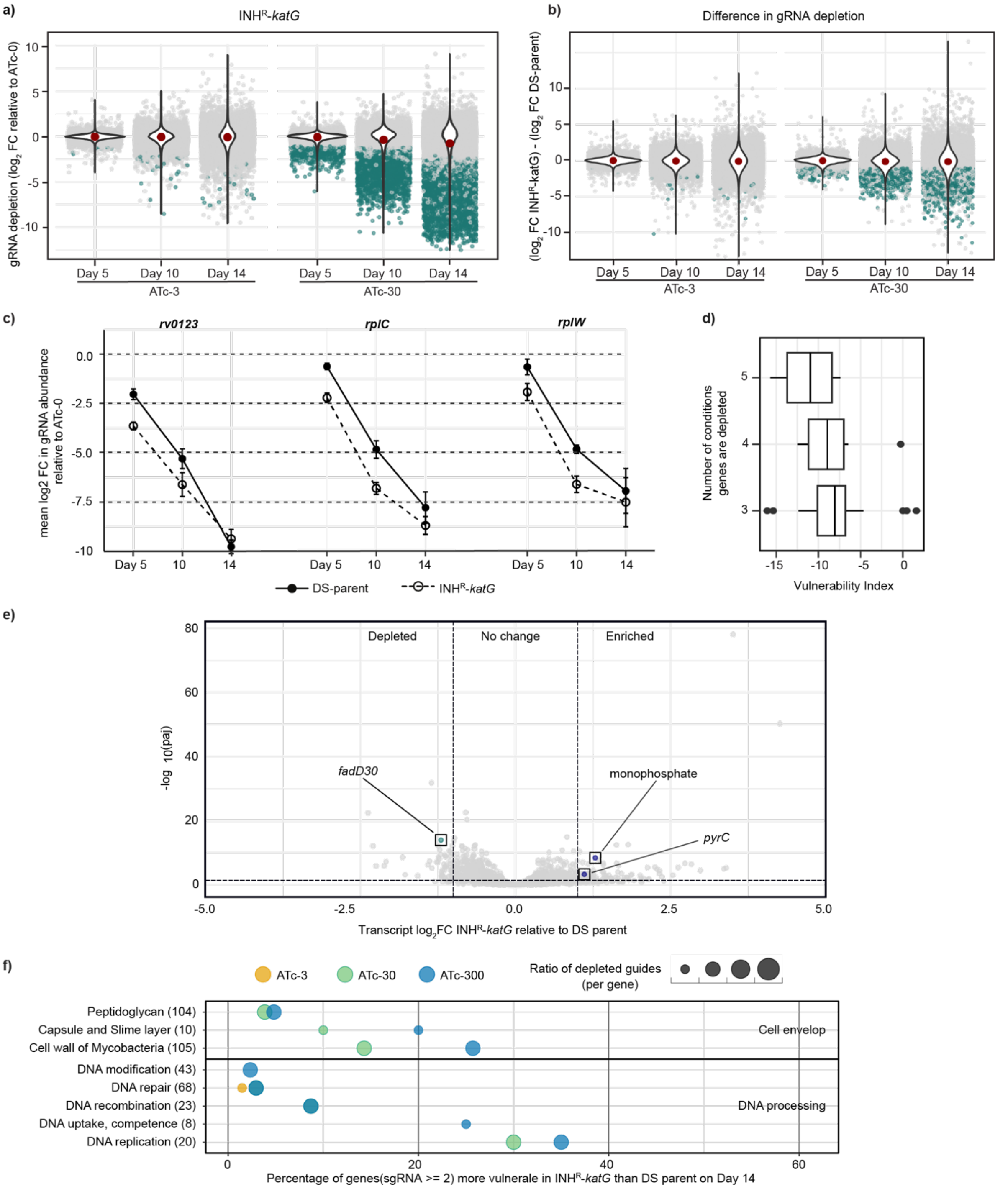

Supplementary Fig. 2| WG-CRISPRi screening to identify genetic vulnerability in INH^R-*katG* *M. tuberculosis* (a-b) The summary of gRNA abundance at ATc-3 and ATc-30 in the *M. tuberculosis* strain (a) INH^R-*katG*, and (b) the depletion difference between the DS-parent and INH^R-*katG*. **(c)** Mean gRNA abundance targeting genes (Rv0123, *rplC* and *rplW*) that were significantly depleted under no less than 3 conditions but not on day 14 ATc-300. Data were plotted as the changes in gRNA abundance relative to its ATc-0 along time points. **(d)** Summary of genes (n =38) that were more depleted under >= 3 conditions but not on day 14+ATc-300. Genes are grouped by the number of depleted conditions and vulnerability index of each gene from CRISPRi-Bosch is plotted on the x-axis. **(e)** Differentially expressed genes in INH^R-*katG*. Each dot represents a single gene. Only the differentially expressed and

more vulnerable genes in INH^R-*katG* are coloured and labelled. **(f)** Pathway analysis of genes that are more vulnerable to inhibition in INH^R-*katG* according to functional subclass. Each panel represents a PATRIC functional class (shown as the annotated text within each panel) broken down into functional subclass classification. Bubble plot represents data from day 14. The x-axis quantifies the proportion of genes called "more vulnerable" over the total number of *M. tuberculosis* genes per functional subclass under cell envelope or DNA processing pathways, and the y-axis shows the name of each functional subclass and the total number of genes in each subclass labelled in brackets. Functional subclasses are described using classifications from the PATRIC database. Within each functional subclass, the dot size indicates the average ratio of gRNAs targeting each gene that is more depleted in INH^R-*katG.* The dot colour denotes the ATc concentration from which the amplicon sequencing was performed. Source data are provided as a Source Data file.

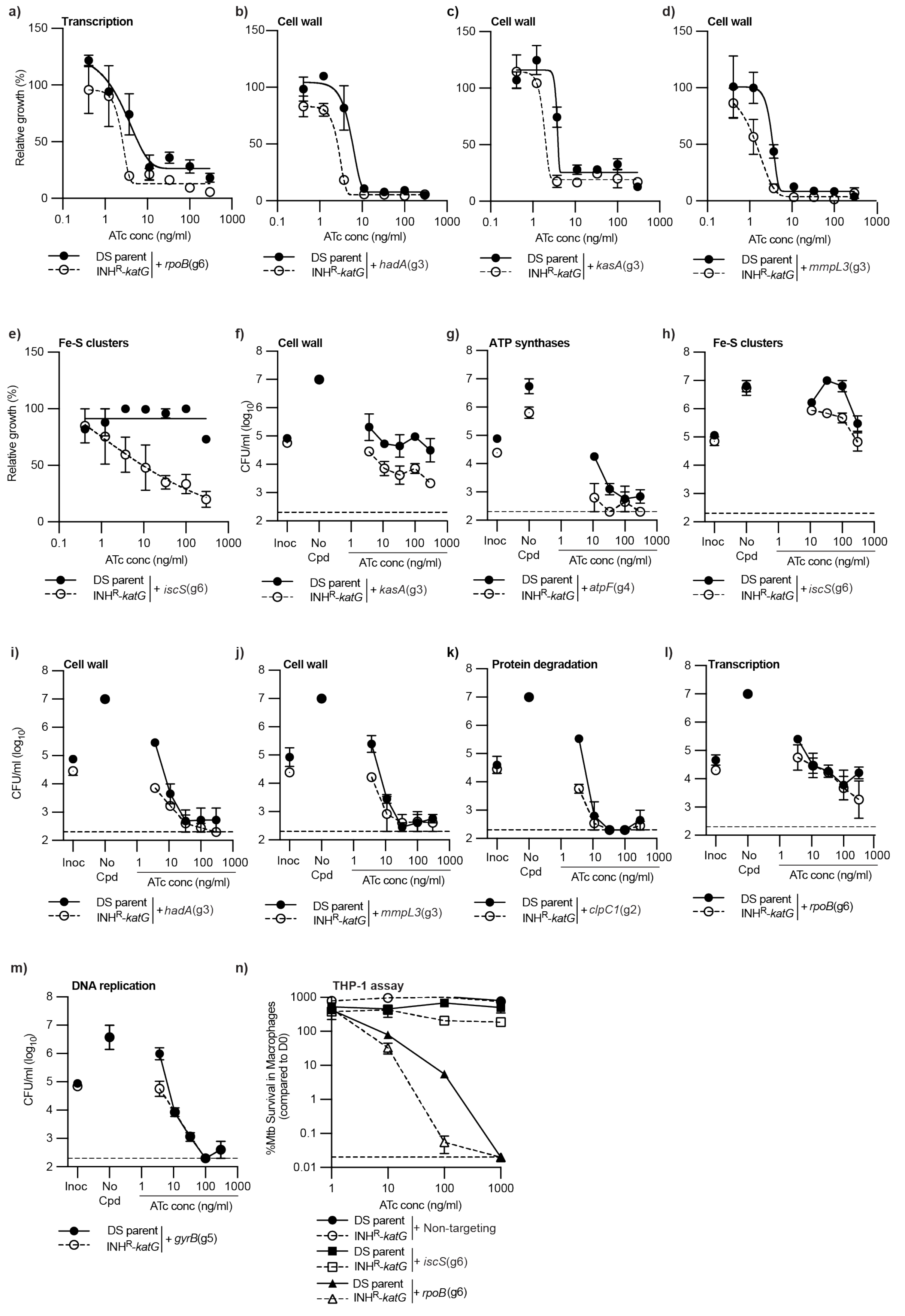

Supplementary Fig. 3| Pathway analysis defines diverse pathways that are more vulnerable to inhibition in INH^{R}-*katG*: (a-e) Growth of *M. tuberculosis* DS-parent

and INH^R-*katG* expressing gRNAs that target (a) *rpoB*, (b) *hadA,* (c) *kasA,* (d) *mmpl3* and (e) *iscS* in ATc dose response assays (mean ± extrema of two biological replicates, n≥3). The (gx) after each gRNA denotes the specific gRNA targeting each gene. (f-m) Viability plots of *M. tuberculosis* DS-parent and INH^R-*katG* expressing for gRNA targeting (f) *kasA,* (g) *atpF,* (h) *iscS,* (i) *hadA,* (j) *mmpl3,* (k) *clpC1*, (l) *rpoB*, and (m) *gyrB.* CFU/ml were determined from 96 well plates at the stated ATc concentrations. Inoc denotes the starting CFU/ml and no-cpd denotes the detected CFU/ml in the absence of ATc (mean ± extrema of two biological replicates, n≥3). Dashed line represents the lower limit of detection. **(n)** THP-1 macrophage cells were infected with *M. tuberculosis* DS-parent and INH^R-*katG* cells expressing gRNAs targeting *iscS*, *rpoB* or with a non-targeting control. CRISPRi was induced with the stated concentrations of ATc and intracellular survival was determined by plating for viable colonies (mean ± SD of three biological replicates, n=2 independent experiments). Source data are provided as a Source Data file.

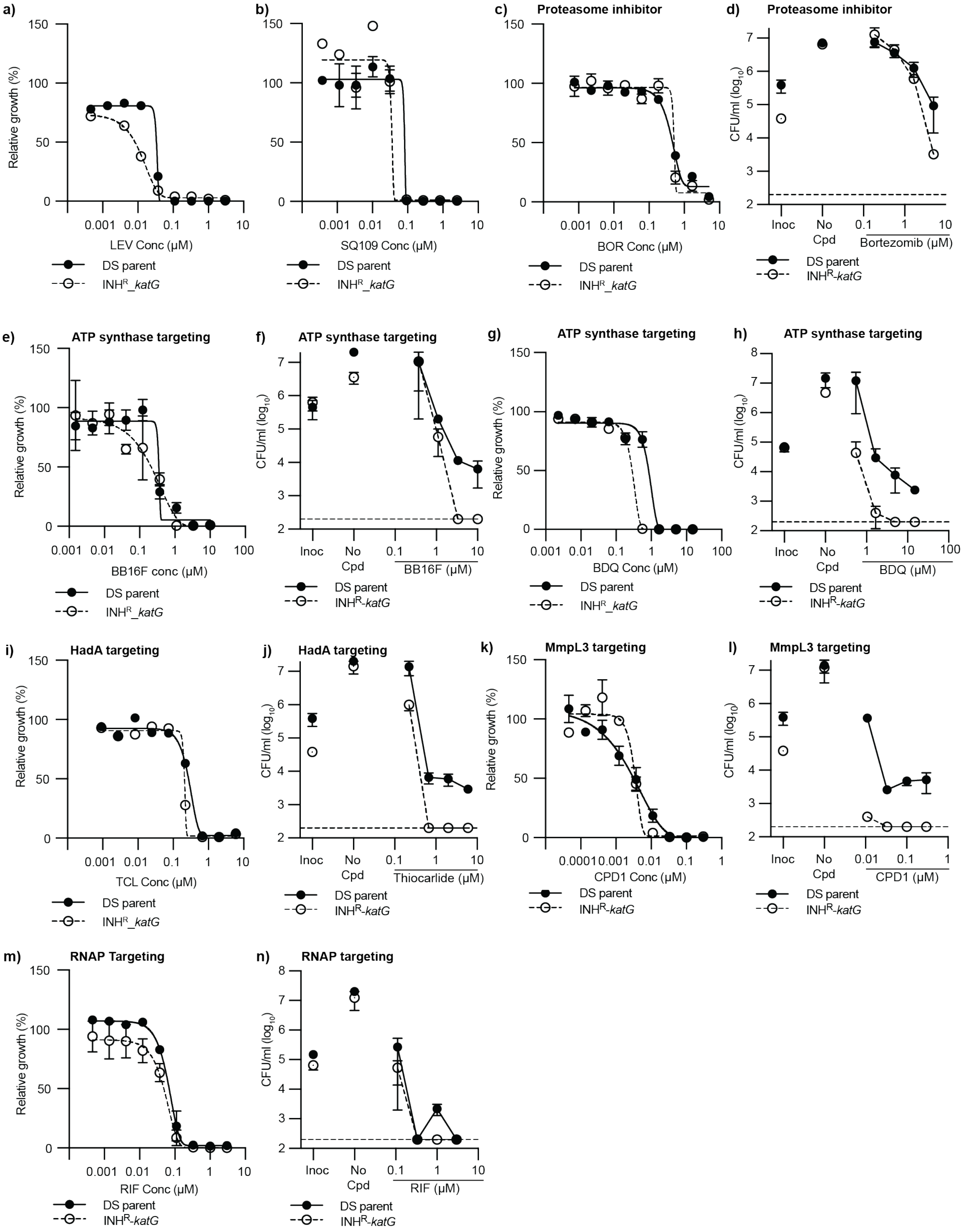

Supplementary Fig. 4| Diverse pathways are more vulnerable to chemical inhibition in INH^R-*katG*: (a-c, e, g, i, k, m) Growth of *M. tuberculosis* DS-parent and INH^R-*katG* (a) levofloxacin, (b) SQ109*,* (c) Bortezomib*,* (e) BB16F, (g) bedaquiline, (i) thiocarlide, (k) CPD1 and (m) rifampicin in dose response assays (mean ± extrema of two biological replicates, n≥3). **(d, f, h, j, l, n)** MBC assays were used to determine the

susceptibility of *M. tuberculosis* DS-parent and INH^R-*katG* to increasing concentrations of (d) Bortezomib*,* (f) BB16F, (h) bedaquiline, (j) thiocarlide, (l) CPD1 and (n) rifampicin. Inoc denotes the starting CFU/ml and no-cpd denotes the detected CFU/ml in the absence of compound (mean ± extrema of two biological replicates, n=3 independent experiments). Dashed line represents the lower limit of detection. Source data are provided as a Source Data file.

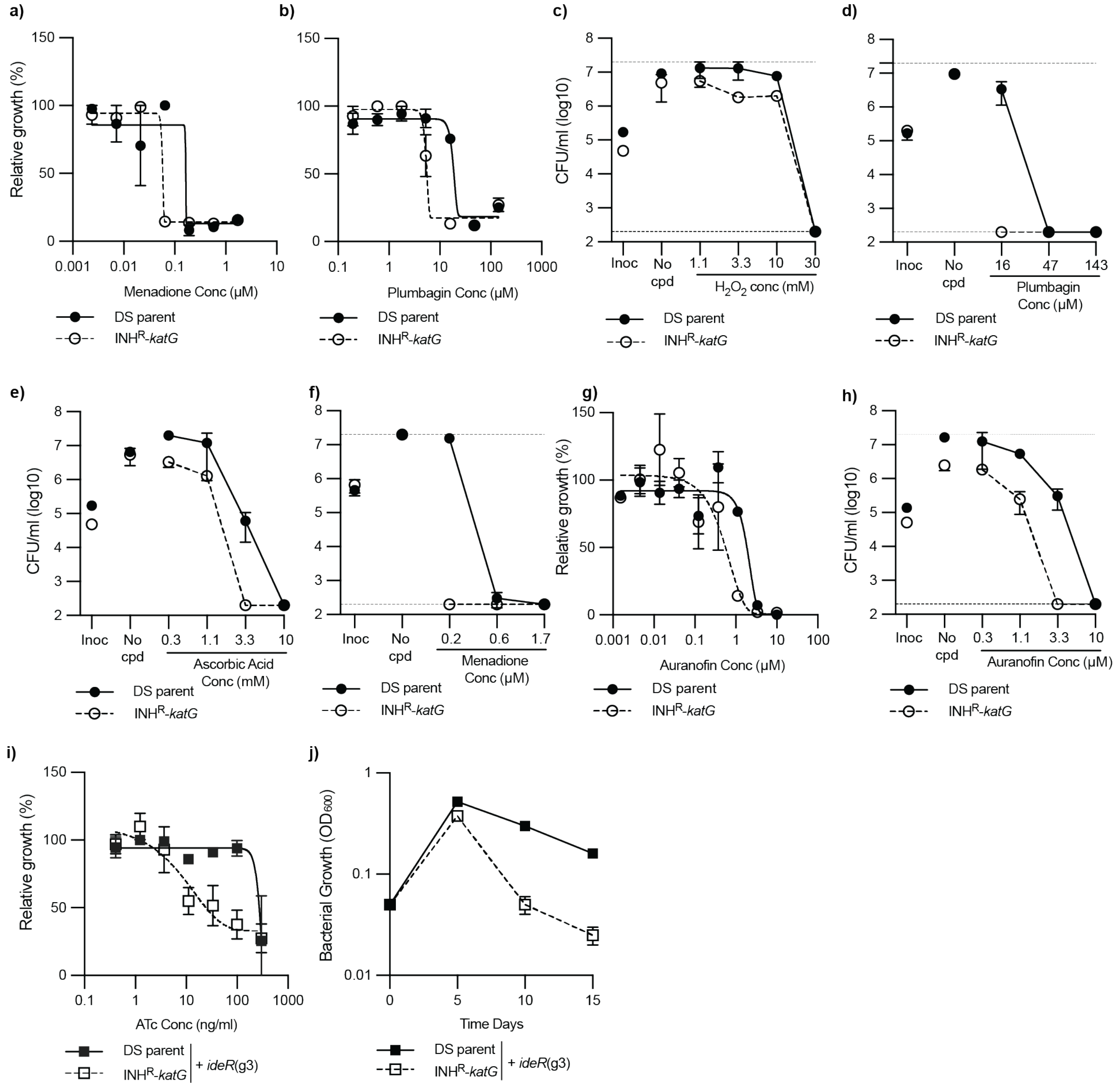

Supplementary Fig. 5| INH^R-*katG* utilizes alternative redox detoxification pathways to compensate for the loss of *katG*: (a-b) Susceptibility of *M. tuberculosis* DS-parent and INH^R-*katG* to growth inhibition by (a) menadione and (b) plumbagin. **(c-f)**. Susceptibility of *M. tuberculosis* DS-parent and INH^R-*katG* to killing by (c) H_2O_2, (d) plumbagin, (e) ascorbic acid, and (f) menadione. **(g-h)** Susceptibility of *M. tuberculosis* DS-parent and INH^R-*katG* to auranofin in 96 well plate dose response assays as detected by (g) growth inhibition and (h) killing (mean ± extrema of two biological replicates, n≥3). Inoc denotes the starting CFU/ml and no-cpd denotes the detected CFU/ml in the absence of compound. Dashed line represents the lower limit of detection. **(i-j)** Growth of *M. tuberculosis* DS-parent and INH^R-*katG* expressing gRNAs that target *ideR* as detected using (i) 96 well plate ATc dose response assays and (j) by maintaining continuous log-phase growth by back diluting 1/20 into fresh media on days 5, 10, 15. All strains were grown in 7H9-K with ATc-300 and growth was determined by measuring OD_{600}. Data is mean ± SD of three biological replicates, (n=2 independent experiments). Source data are provided as a Source Data file.

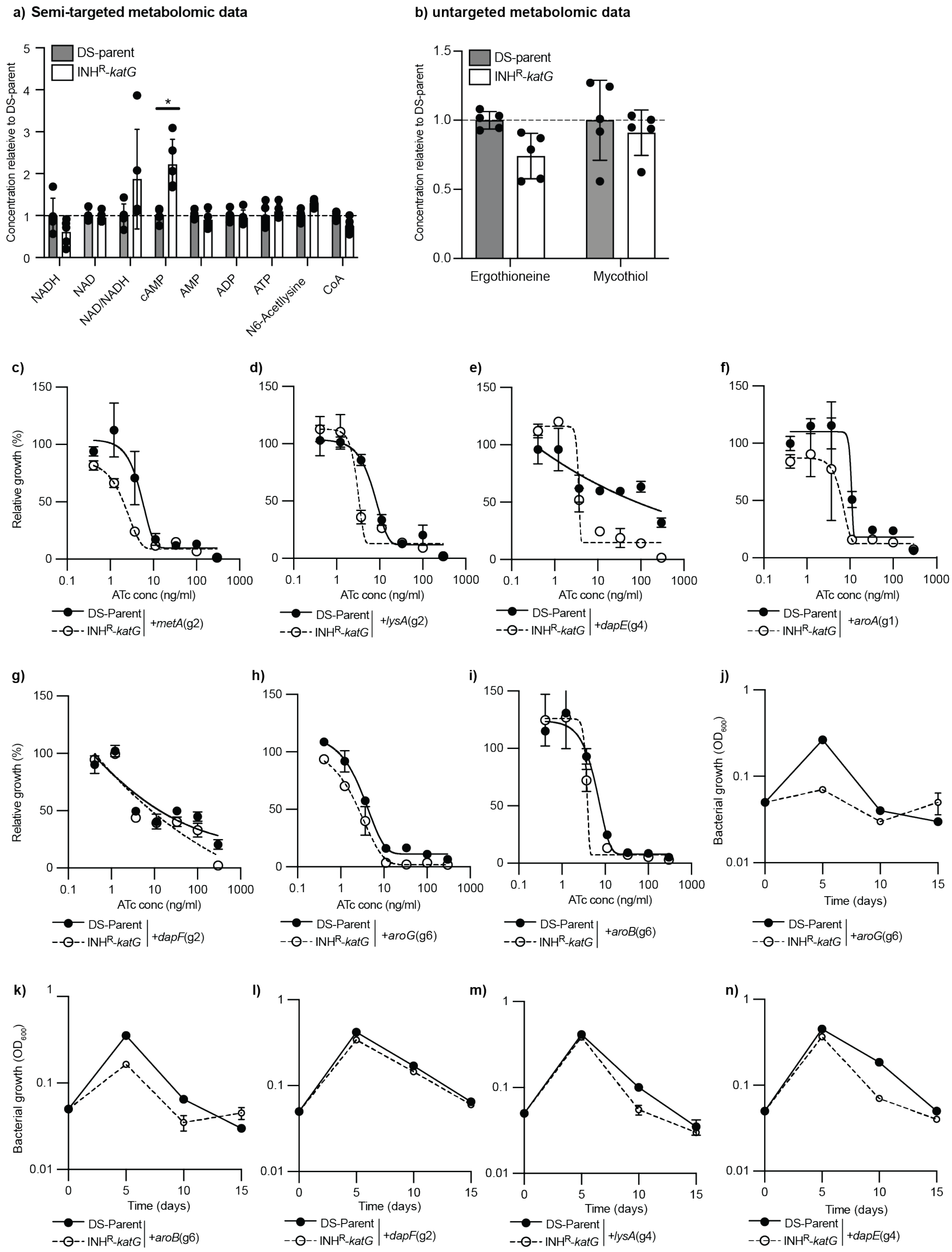

Supplementary Fig. 6| Amino acid metabolism is altered in an INH^{R}-*katG* mutant **(a-b)** Changes in the relative abundance of metabolites were determined from five replicates. Metabolites with an asterisk "*" are those that are significantly depleted (i.e. ≥1.5 foldchange in peak measures and p-value <0.05 as determined by two way ANOVA). For (b) peak measures were determined using predicted masses for ergothioneine and mycothiol. **(c-i)** Growth of *M. tuberculosis* DS-parent and INH^{R}-*katG*

expressing gRNA targeting (c) *metA*, (d) *lysA*, (e) *dapE*, (f) *aroA*, (g) *dapF*, (h) *aroG*, and (i) *aroB* in ATc dose response assays (mean ± extrema of two biological replicates, n≥3). The (gx) after each gRNA denotes the specific gRNA targeting each gene. **(j-n)** Growth kinetics of *M. tuberculosis* DS-parent and INH^{R}-*katG* expressing a gRNA targeting (j) *aroG*, (k) *aroB*, (l) *dapF*, (m) *lysA*, and (n) *dapE*. Bacterial growth was measured by OD_{600} and back diluted 1/20 into fresh media on days 5, 10, 15. All strains were grown in 7H9-K ATc-300. Source data are provided as a Source Data file.

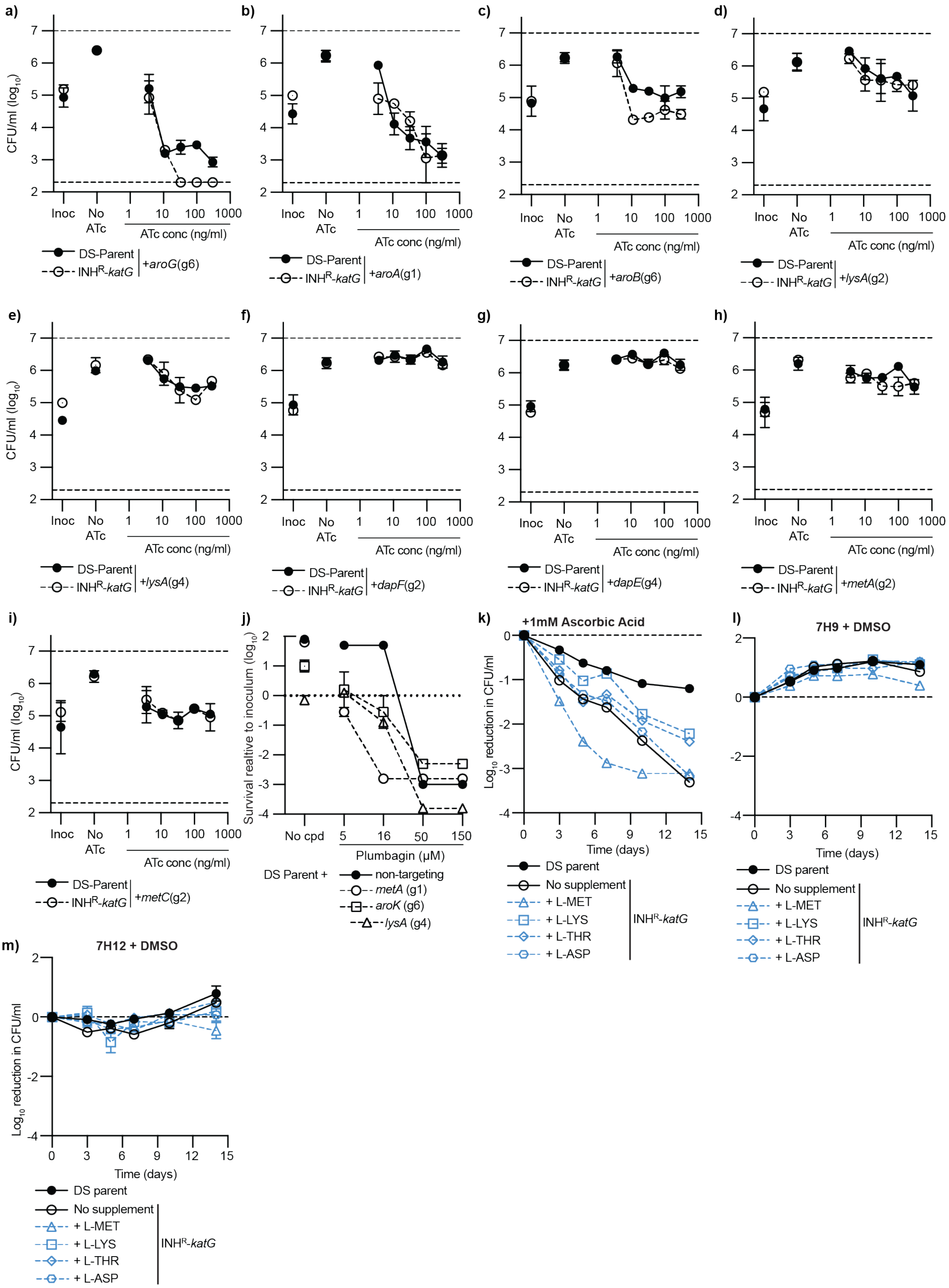

Supplementary Fig. 7| Amino acid metabolism is altered in a INH^{R}-*katG* mutant (a-i) Viability plots of *M. tuberculosis* DS-parent and INH^{R}-*katG* expressing for gRNA targeting (a) *aroG*, (b) *aroA*, (c) *aroB*, (d-e) *lysA*, (f) *dapF*, (g) *dapE*, (h) *metA*, and (i) *metC*. CFU/ml was determined from 96 well plates. Inoc denotes the starting CFU/ml and no-cpd denotes the detected CFU/ml in the absence of ATc (mean ± extrema of

two biological replicates, n≥3). Dashed line represents the lower limit of detection. **(j)** The DS-parent of *M. tuberculosis* mc^{2}6206 was pre-depleted for *metA*, *aroK* and *lysA* for 5 days, prior to exposure to plumbagin at the stated concentrations. No-cpd represents the viability of the pre depleted strains in the absence of compound but in the presence of 300 ng/ml ATc. Data is expressed as the reduction in viable colonies on day 10, relative to the starting inoculum on day 0. A non-targeting gRNA is included as a negative control. **(k-m)** The DS-parent or INH^R-*katG* were grown in either (k) 7H12 media with glucose as a sole carbon with 1 mM ascorbic acid, (l) 7H9 supplemented with OADC and DMSO or (m) 7H12 media with glucose as a sole carbon with DMSO and the stated amino acids at the following concentrations. (i.e. L-methionine (20 µg/ml), L-lysine (50 µg/ml), L-threonine (50µg/ml) and L-asparate (5 µg/ml). Data is expressed as the reduction in viable colonies at each time point relative to the starting inoculum on day 0. Source data are provided as a Source Data file.

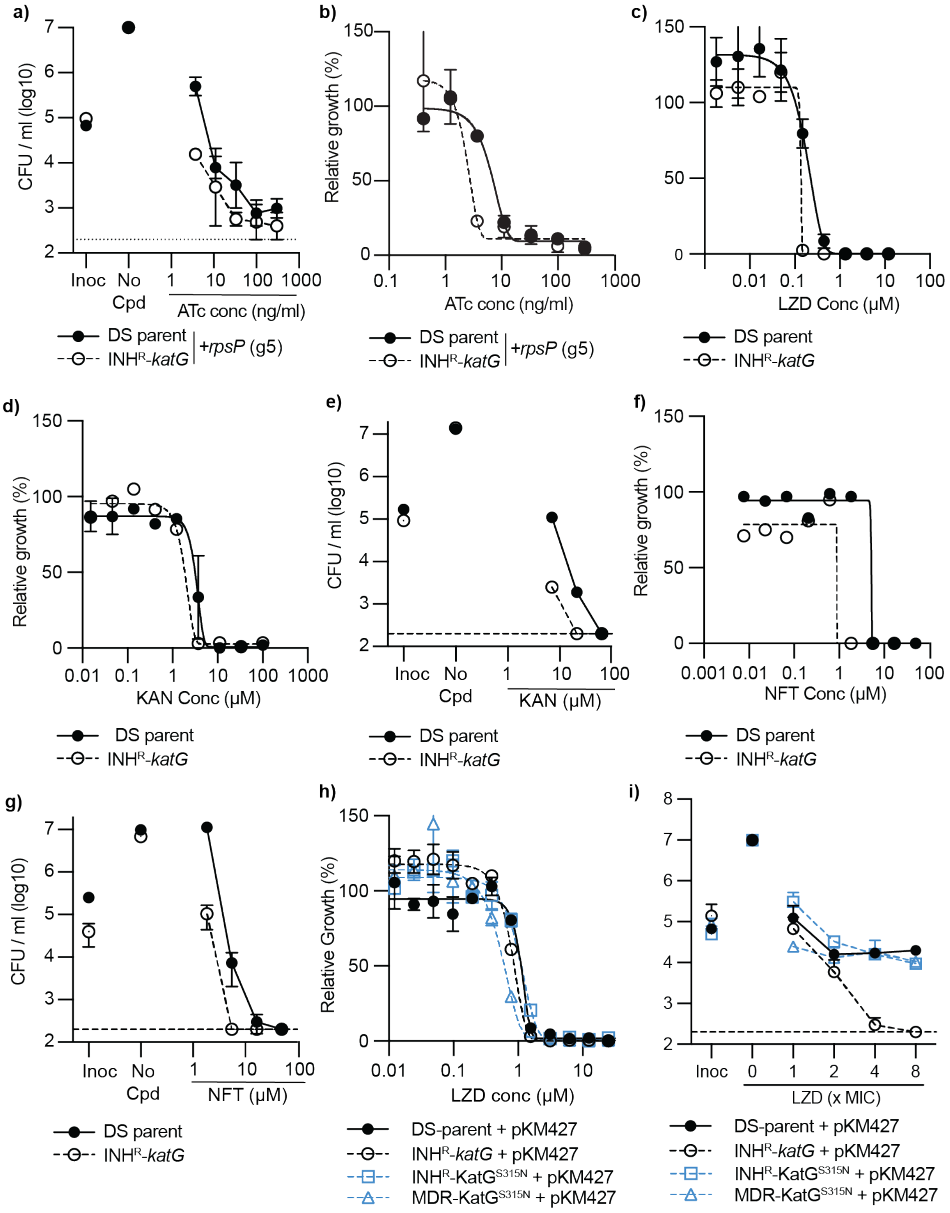

Supplementary Fig. 8| Ribosome biogenesis is more vulnerable to inhibition in an INH^R-*katG* mutant: (a) Viability plots of *M. tuberculosis* DS-parent and INH^R-*katG* expressing for gRNA targeting *rpsP.* CFU/ml were determined from 96 well plates at the stated ATc concentrations.**(b)** Growth of *M. tuberculosis* DS-parent and INH^R-*katG* expressing gRNAs targeting *rpsP* in ATc dose response assays (mean ± extrema of two biological replicates, n≥3). The (gx) after each gRNA denotes the specific gRNA targeting each gene. **(c-g)** Susceptibility of *M. tuberculosis* DS-parent and INH^R-*katG* to increasing concentrations of (c) linezolid, (d-e) kanamycin, and (f-g) nitrofurantoin. Inoc denotes the starting CFU/ml and no-cpd denotes the detected CFU/ml in the absence of compound or ATc (mean ± extrema of two biological replicates, n=3 independent experiments). Dashed line represents the lower limit of detection. **(h-i)** Susceptibility of *M. tuberculosis* DS-parent, INH^R-*katG*, INH^R-KatG^{S315N} and a MDR-KatG^{S315N} + RpoB^{D435V} strains to inhibition and killing by linezolid. Both the MIC assays and MBC assays are mean ± extrema of two biological replicates, n=2 independent

experiments. For MBC assays Inoc denotes the starting CFU/ml and no-cpd denotes the detected CFU/ml in the absence of ATc. The dashed line represents the lower limit of detection. All strains contain the chromosomally integrated plasmid pKM427 that is used as part of mycobacterial recombineering. The LZD MIC is 1.5 μM as determined in h).
