# Supplementary material for: Whole genome CRISPRi screening identifies druggable vulnerabilities in an isoniazid resistant strain of Mycobacterium tuberculosis
Source: Nat Commun. 2024 Nov 13;15:9791. doi: 10.1038/s41467-024-54072-w (PMC11560980; doi:10.1038/s41467-024-54072-w)
Supplement: Supplementary file 3 — Description of additional supplementary files [file 41467_2024_54072_MOESM3_ESM.pdf]

Description of Additional Supplementary Files

Supplementary Data 1 - List of guides used in this study

Supplementary Data 2 - Comparison of essential calls between this study and Bosch 2021

Supplementary Data 3 - List of differentially expressed genes identified in INHR-katG relative to the DS-parent (RNAseq data)

Supplementary Data 4 - Pathway analyses of genes classified into functional subclasses. Functional subclasses are described using classifications from the PATRIC database.

Supplementary Data 5 - Median normalised peaks heights of each metabolite detected for INHR-katG and the DS-parent.

Supplementary Data 6 - List of primers used in this study

Supplementary Data 7 - List of plasmids used in this study
