# Supplementary material for: Whole genome CRISPRi screening identifies druggable vulnerabilities in an isoniazid resistant strain of Mycobacterium tuberculosis
Source: Nat Commun. 2024 Nov 13;15:9791. doi: 10.1038/s41467-024-54072-w (PMC11560980; doi:10.1038/s41467-024-54072-w)
Supplement: Supplementary file 5 — Reporting summary [file 41467_2024_54072_MOESM5_ESM.pdf]

nature portfolio

Corresponding author(s): Matthew McNeil

Last updated by author(s): Oct 16, 2024

Reporting Summary

Nature Portfolio wishes to improve the reproducibility of the work that we publish. This form provides structure for consistency and transparency in reporting. For further information on Nature Portfolio policies, see our Editorial Policies and the Editorial Policy Checklist.

Statistics

For all statistical analyses, confirm that the following items are present in the figure legend, table legend, main text, or Methods section.

n/a	Confirmed	
☐	☒	The exact sample size (*n*) for each experimental group/condition, given as a discrete number and unit of measurement
☐	☒	A statement on whether measurements were taken from distinct samples or whether the same sample was measured repeatedly
☐	☒	The statistical test(s) used AND whether they are one- or two-sided<br>*Only common tests should be described solely by name; describe more complex techniques in the Methods section.*
☒	☐	A description of all covariates tested
☐	☒	A description of any assumptions or corrections, such as tests of normality and adjustment for multiple comparisons
☐	☒	A full description of the statistical parameters including central tendency (e.g. means) or other basic estimates (e.g. regression coefficient) AND variation (e.g. standard deviation) or associated estimates of uncertainty (e.g. confidence intervals)
☐	☒	For null hypothesis testing, the test statistic (e.g. *F*, *t*, *r*) with confidence intervals, effect sizes, degrees of freedom and *P* value noted<br>*Give P values as exact values whenever suitable.*
☒	☐	For Bayesian analysis, information on the choice of priors and Markov chain Monte Carlo settings
☐	☒	For hierarchical and complex designs, identification of the appropriate level for tests and full reporting of outcomes
☒	☐	Estimates of effect sizes (e.g. Cohen's *d*, Pearson's *r*), indicating how they were calculated

Our web collection on statistics for biologists contains articles on many of the points above.

Software and code

Policy information about availability of computer code

Data collection	Semi-targeted metabolomic data was collected using Thermo Tracefinder (V 4.1) (General Quan Browser).
Data analysis	CRISPRi data analyses<br>Demultiplexed fastq files were converted to tabular format using SeqKit (v2.3.1). Differential abundance tests were performed via edgeR (v3.42.4). R (v4.3.0) was used to develop customised scripts (available from GitHub [https://github.com/Cecilia-Wang/2023_CRISPRI]) to determine genes with increased vulnerability.<br>Metabolomics<br>Metabolites were assigned to sample peaks in El-Maven v.0.12.1 Raw data were processed and analysed using the MetaboAnalyst v5.0 web server (https://www.metaboanalyst.ca/docs/About.xhtml) using the Statistical Analysis [one factor] module.<br>RNA-seq<br>Demultiplexing, quality control, and adapter trimming was performed with bcl-convert (v3.9.3). Adaptor removal and quality trimming were conducted with bbduk (a part of the BBTools suite bbmap v39.06 https://sourceforge.net/projects/bbmap/). The cleaned paired-end transcriptomic FASTQ files were then aligned to the Mycobacterium tuberculosis mc26206 complete genome (NCBI Accession number: PRJNA914416) with Bowtie2 using the default settings (v2.4.5). The output alignments were saved as SAM files, converted to sorted BAM files, and produced index BAI files with SAMtools (v1.16.1). The resulting alignment files (i.e. BAM and BAI files) were loaded in R (v4.3.0) with the package Rsamtools (v2.16.0). Gene counts were calculated using packages GenomicFeatures (v1.52.0) and GenomicAlignments (v1.36.0). Differential expression of each gene was calculated with DESeq2 (v1.40.1) between DS-parent and INHR-katG strains.<br><br>Misc<br>Microsoft Excel

Geneious Prime
GraphPad Prism (Version 10)

For manuscripts utilizing custom algorithms or software that are central to the research but not yet described in published literature, software must be made available to editors and reviewers. We strongly encourage code deposition in a community repository (e.g. GitHub). See the Nature Portfolio guidelines for submitting code & software for further information.

Data

Policy information about availability of data

All manuscripts must include a data availability statement. This statement should provide the following information, where applicable:

- Accession codes, unique identifiers, or web links for publicly available datasets
- A description of any restrictions on data availability
- For clinical datasets or third party data, please ensure that the statement adheres to our policy

The CRISPRi results sequencing data and RNA sequencing data that support the findings of this study have been deposited in NCBI Sequence Read Archive with the BioProject accession code PRJNA1041353 [https://www.ncbi.nlm.nih.gov/bioproject/?term=PRJNA1041353]. The Metabolomic data generated in this study have been deposited in the MetaboLights database under accession code MTBLS11277 [https://www.ebi.ac.uk/metabolights/editor/MTBLS11277/descriptors]. Manually curated pathway calls were derived from the PATRIC databases [https://www.bv-brc.org/search/?and(keyword(Mycobacterium),keyword(tuberculosis),keyword(H37Rv))]. Source data are provided with this paper.

Research involving human participants, their data, or biological material

Policy information about studies with human participants or human data. See also policy information about sex, gender (identity/presentation), and sexual orientation and race, ethnicity and racism.

Reporting on sex and gender	not applicable
Reporting on race, ethnicity, or other socially relevant groupings	not applicable
Population characteristics	not applicable
Recruitment	not applicable
Ethics oversight	not applicable

Note that full information on the approval of the study protocol must also be provided in the manuscript.

Field-specific reporting

Please select the one below that is the best fit for your research. If you are not sure, read the appropriate sections before making your selection.

☒ Life sciences ☐ Behavioural & social sciences ☐ Ecological, evolutionary & environmental sciences

For a reference copy of the document with all sections, see nature.com/documents/nr-reporting-summary-flat.pdf

Life sciences study design

All studies must disclose on these points even when the disclosure is negative.

Sample size

No statistical method was used to predetermine the sample size.

Data exclusions

CRISPRi data:
One DS-parent replicate out of five taken on day 5 ATc-0 was destroyed due to contamination caused by a technical issue, and thus was not included in the study.

Experimental data:
For MIC data, data points >150% the no compound control were excluded when then were flanked by data points that were between 75-150% of the no compound control.

No data exclusion for RNA-seq analyses

Metabolomic data:
One of the six replicates in the metabolomic data was identified as an outlier and was excluded from the analysis.

Replication

Five replicates were used for both the CRISPRi screen and metabolomic data analyses.
For experimental validations, where small number of replicates (2 to 3) were used due to practicality issues, however multiple attempts were conducted and were successful.

Randomization	The experiments were not randomized.
Blinding	The Investigators were not blinded to allocation during experiments and outcome assessment.

Reporting for specific materials, systems and methods

We require information from authors about some types of materials, experimental systems and methods used in many studies. Here, indicate whether each material, system or method listed is relevant to your study. If you are not sure if a list item applies to your research, read the appropriate section before selecting a response.

Materials & experimental systems

n/a	Involved in the study
☒	☐ Antibodies
☐	☒ Eukaryotic cell lines
☒	☐ Palaeontology and archaeology
☒	☐ Animals and other organisms
☒	☐ Clinical data
☒	☐ Dual use research of concern
☒	☐ Plants

Methods

n/a	Involved in the study
☒	☐ ChIP-seq
☒	☐ Flow cytometry
☒	☐ MRI-based neuroimaging

Eukaryotic cell lines

Policy information about cell lines and Sex and Gender in Research

Cell line source(s)	THP-1 is a monocyte isolated from peripheral blood of an acute leukemia patient. THP-1 cell line was obtained from ATCC (Cat# TIB-202)
Authentication	Cell lines were not authenticated
Mycoplasma contamination	Cell lines were not tested for mycoplasma
Commonly misidentified lines (See ICLAC register)	No commonly misidentified cell lines were used in this study.

Plants

Seed stocks	not applicable
Novel plant genotypes	not applicable
Authentication	not applicable
